# Arrangement at the nanoscale: Effect on magnetic particle hyperthermia

**DOI:** 10.1038/srep37934

**Published:** 2016-11-29

**Authors:** E. Myrovali, N. Maniotis, A. Makridis, A. Terzopoulou, V. Ntomprougkidis, K. Simeonidis, D. Sakellari, O. Kalogirou, T. Samaras, R. Salikhov, M. Spasova, M. Farle, U. Wiedwald, M. Angelakeris

**Affiliations:** 1 Physics Department, Aristotle University of Thessaloniki, Thessaloniki, 54124, Greece; 2 Faculty of Physics and Center for Nanointegration Duisburg-Essen (CENIDE), University of Duisburg-Essen, 47048 Duisburg, Germany

## Abstract

In this work, we present the arrangement of Fe_3_O_4_ magnetic nanoparticles into 3D linear chains and its effect on magnetic particle hyperthermia efficiency. The alignment has been performed under a 40 mT magnetic field in an agarose gel matrix. Two different sizes of magnetite nanoparticles, 10 and 40 nm, have been examined, exhibiting room temperature superparamagnetic and ferromagnetic behavior, in terms of DC magnetic field, respectively. The chain formation is experimentally visualized by scanning electron microscopy images. A molecular Dynamics anisotropic diffusion model that outlines the role of intrinsic particle properties and inter-particle distances on dipolar interactions has been used to simulate the chain formation process. The anisotropic character of the aligned samples is also reflected to ferromagnetic resonance and static magnetometry measurements. Compared to the non-aligned samples, magnetically aligned ones present enhanced heating efficiency increasing specific loss power value by a factor of two. Dipolar interactions are responsible for the chain formation of controllable density and thickness inducing shape anisotropy, which in turn enhances magnetic particle hyperthermia efficiency.

Magnetic particle hyperthermia (MPH) is a synergistic cancer treatment technique that takes advantage of heat release by magnetic nanoparticles (MNPs) when exposed to an alternating magnetic field in the 100–1000 kHz region. This excitation leads cancer cells either to a serious thermal shock or even to cell death^1^. The heating efficiency of MNPs is quantified by the specific loss power (SLP) value determined as the power dissipation per unit mass of the magnetic material. The SLP magnitude depends on different parameters, such as the intrinsic magnetic properties (e.g. magnetization and magnetic anisotropy), the features of the dispersing medium (e.g. viscosity, stability and concentration) and the field magnitude (amplitude and frequency)^2^.

MNPs, in general, may appear either as isolated nano-entities such as nano-spheres^3^, -cubes^4^, hexagonal polyhedrons^5^, or form larger aggregates such as rings^6^, or nanowires due to dipolar interactions between the particles^7^. The configuration of MNPs in 2D or even 3D chains, has been proposed as an alternative way to control their macroscopic magnetic response on demand^8^,^9^,^10^, Such chains directly affect the hysteresis losses^11^, the effective anisotropy^12^ and susceptibility^13^. Mobility of the MNPs within the colloidal dispersion is another important factor that affects the chain formation success rate, since diverse effects, such as magnetic, electrostatic, viscous, gravitational and molecular interactions are involved^14^. Magnetic dipole-dipole interaction preferably leads to local arrangements at zero field or to chains under an appropriate external magnetic field drive^15^,^16^, Consequently, the controlled assembly of MNPs into chains inducing shape anisotropy along the alignment axis may open an alternative way to optimized heating efficiencies^17^.

Depending on conditions, MNPs may arrange randomly or in order, according to the complex three-dimensional magnetic dipolar coupling between them in conjunction with external field interaction^18^. The formation of chain arrays can be realized through the dispersion of MNPs in a gel matrix^19^. The viscosity of the system may be adjusted by the choice of the matrix material, dispersion concentration or temperature. A good gel matrix candidate is agarose, one of the two major components of the polygalactoside agarose, with the second one being agaropectine. Agarose gelation takes place only when constituent microstructures form ordered regions (i.e. junctions)^20^. Remarkably, previous experimental studies outlined that a 3–4% agarose gel content has a microstructure similar to hard tissues^21^ while lower content gels have porosities similar to soft tissues such as the human brain^22^ mimicking in both cases different tissue properties.

In this work, we prepared Fe_3_O_4_ MNPs chains in an agarose gel matrix and tested them for enhanced magnetic particle hyperthermia efficiency against reference random dispersions. For this purpose, 10 nm and 40 nm MNPs were dispersed in hot agarose solution followed by cool-down gelation to ambient temperature under a static magnetic field. We experimentally evaluate optimum parameters and conditions for successful chain formation by tuning dipolar interactions, based on intrinsic magnetic profile of constituent particles, dispersion concentration and agarose content. The formation of chains is proven by scanning electron microscopy (SEM) images and ferromagnetic resonance (FMR) spectroscopy. Simulation performed using an anisotropic molecular dynamics model is in good agreement with the experimental results. Successful chain formation results to increased SLP values by about a factor of two in the case of the 40 nm Fe_3_O_4_ MNPs, as quantified by calorimetric experiments. The enhanced heating efficiency results are successfully correlated to magnetic property theoretical calculations in the frame of a modified Stoner-Wohlfarth model taking into account the temperature and frequency dependence of the coercive field.

## Results and Discussion

**Figure 1:**
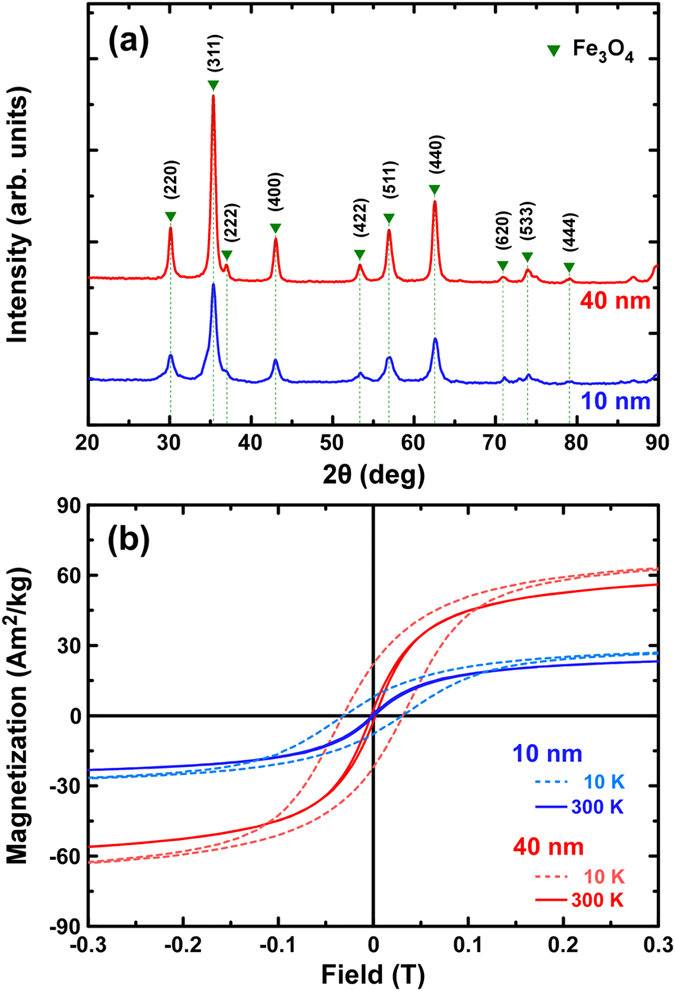
Structural (**a**) and magnetic (**b**) characterization of powder samples of 10 and 40 nm Fe_3_O_4_ MNPs. Green triangles indicate the reference peak positions of bulk magnetite.

Structural and magnetic characterization of the 10 and 40 nm powder Fe_3_O_4_ constituent MNPs was performed. The corresponding XRD graphs are shown in Fig. 1a where the typical reflections of magnetite were detected for both samples indicating the presence of single phase magnetite structure. 10 nm MNPs present wider peaks’ width together with lower intensities as expected from their smaller crystallite size.

Hysteresis loops were recorded at 10 K and 300 K (Fig. 1b) followed by a zero-field cooling/field cooling (ZFC/FC) experiment at 5 mT displayed in Fig. S1 of the supporting information. At room temperature, 40 nm MNPs present a typical ferromagnetic (FM) hysteresis loop while 10 nm MNPs show almost zero coercivity resembling superparamagnetic (SPM) behavior in terms of static magnetic field at 300 K. At low temperatures (10 K), both samples show ferromagnetic behavior. Due to their small size, 10 nm MNPs present much lower saturation magnetization. The corresponding ZFC/FC curves confirm the different room temperature magnetic behavior of the two particle systems (Fig. S1). It should be mentioned, that the magnetic variations, between the two systems, due to size effects, appear suppressed due to aggregation effects apparent in powder samples composed of MNPs synthesized by the proposed methodology (at absence of surfactants) as described in Methods section.

**Figure 2:**
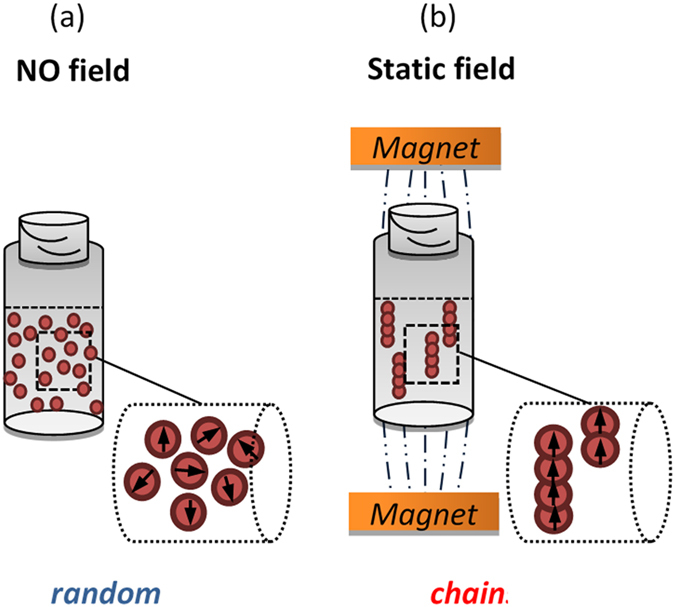
Schematic representation of (**a**) reference sample (random), where the MNPs are fixed at random positions in the agarose matrix and (**b**) magnetically aligned sample with MNPs’ chain formation (chain) in an external field of 40 mT.

From the as synthesized 10 and 40 nm Fe_3_O_4_ powders, we prepared chain samples, by dispersing them in an agarose solution of varying content, 1–20 mg/mL (Fig. 2). When an external magnetic field is present, the particles are subjected to an attractive force along the field direction and a repulsive force normal to it. When the particles are allowed to aggregate, in the presence of the external field, linear aggregates are formed due to the anisotropic character of the inter-particle dipolar interactions. With the axes of the pearl-chain-like clusters aligned along the field direction. At relatively high particle concentrations, even more complex structures may arise. Another effect that should not be neglected a priori is the interaction occurring between chain forming particles. At weak field strengths, the degree of magnetization of the particles is proportional to the local field strength and so, the net magnetization of the particles contained within a chain is enhanced by the presence of neighboring particles. This effect leads to an increased range of the magnetic interaction between the aggregates as they gain in size.

A simple setup of an external magnetic field of 40 mT was used to align the MNPs in linear chains in an agarose gel (Fig. S2a) while in Figs S2b–S2f the optimization of parameters used for chain formation are graphically illustrated. After natural cooling down, the chains are quenched in agarose. For each chain-sample, a reference random-sample was also prepared at the absence of the external magnetic field (Fig. 2).

**Figure 3:**
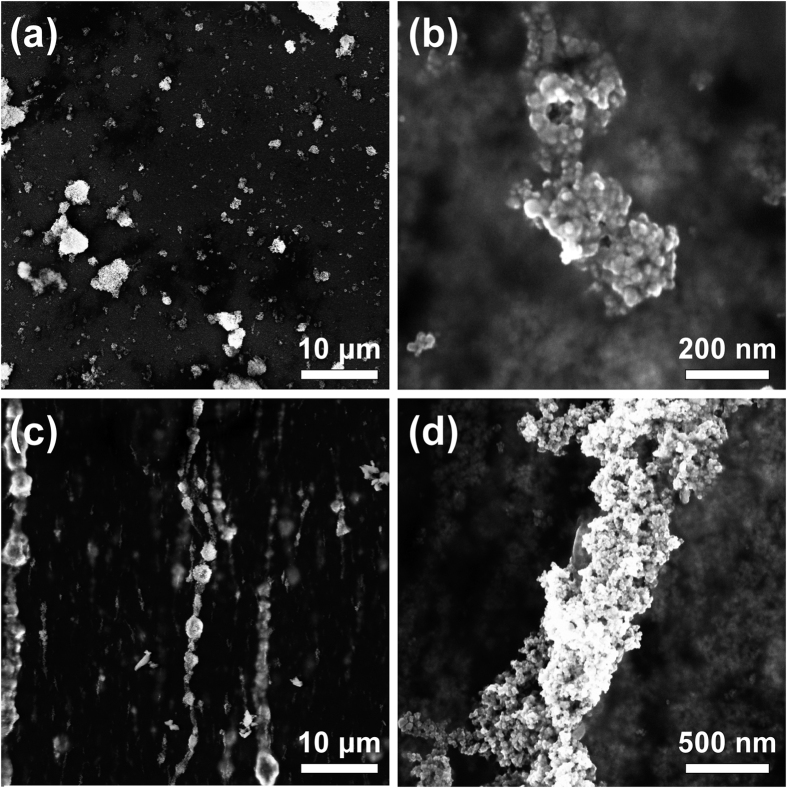
Typical SEM images for 40 nm MNPs with 2 mg/mL concentration and 1 mg/mL agarose content at lower and higher magnification: (**a,b**) random sample and (**c,d**) chain sample.

The microstructure of aggregates may be visualized by SEM. An example is illustrated in Fig. 3 for 40 nm sample with MNPs concentration of 2 mg/mL and agarose content of 1 mg/mL. Figure 3a,b present the SEM images of the random sample at different magnifications as solid aggregates of arbitrary size and shape. Figure 3c shows SEM images of the magnetically aligned samples where linear chains appear relatively uniform in size and density throughout the imaged area. Micrographs were taken at different sample areas revealing homogeneity over the entire sample. Figure 3d depicts a SEM image at higher magnification of a typical chain, to further investigate their morphological details. It is apparent from a series of such images of varying particle and agarose contents, that, depending on particle concentration, chains are well separated from each other and each chain is practically an “oriented” MNPs’ aggregate.

The corresponding images for the SPM sample of 10 nm are presented in Figure S3 where the particles show a minor tendency for chain formation after exposed in an external field. This behavior is attributed to the much smaller dipolar interactions between the 10 nm MNPs in the agarose gel^23^.

The comprehension of the field-driven thermally activated processes in MNPs is still among the cornerstones of application developments in biomedicine. Thermal fluctuations introduce new intrinsic time scales competing with those of external driving forces necessitating a consistent out of-equilibrium thermodynamic theory, applicable to MNPs, which radically complicates the design and optimization procedures in practice. For a ‘heat assisted’ (i.e. MPH) cancer treatment modality, quantification of the specific heating rate in a system comprised of MNPs with statistically distributed properties, such as those envisaged, remains an open problem. Since, hysteresis is proposed as the dominant mechanism giving rise to heating, the assumptions of small driving field amplitudes and negligible inter-particle interactions should be reconsidered to go beyond linear response theory^24^,^25^,^26^.

To theoretically simulate the chain formation process in MNPs’ colloidal suspensions we implemented a 3D Molecular Dynamics (MD) methodology based on an *on-the-fly* coarse-grain (CG) model^27^ following the need to account for microscopic time and length scales but also reach macroscopic time scales at low computational cost. In this frame, simulation starts with (a) the motion of MNPs and (b) the interaction between individual MNPs. As the simulation advances, MNPs form chains due to the magnetic dipole-dipole attraction induced by the external magnetic field. The arrangement of the CG objects (single MNPs or aggregates) is dictated by (a) an anisotropic diffusion model and (b) an effective short-range interaction between CG objects determined by the magnetic dipole-dipole interaction. The term *on the fly* refers to the fact that, the CG objects of the simulation are not fixed *a priori* at the beginning of the simulation but are redefined during its duration. Consequently, the number of CG objects decreases with time and the simulation speeds up as the time advances, allowing calculation resolution adjustments and much longer simulation runs yet requiring less computer power. Details of MD simulation are included in Methods section and Supporting Information section.

**Figure 4:**
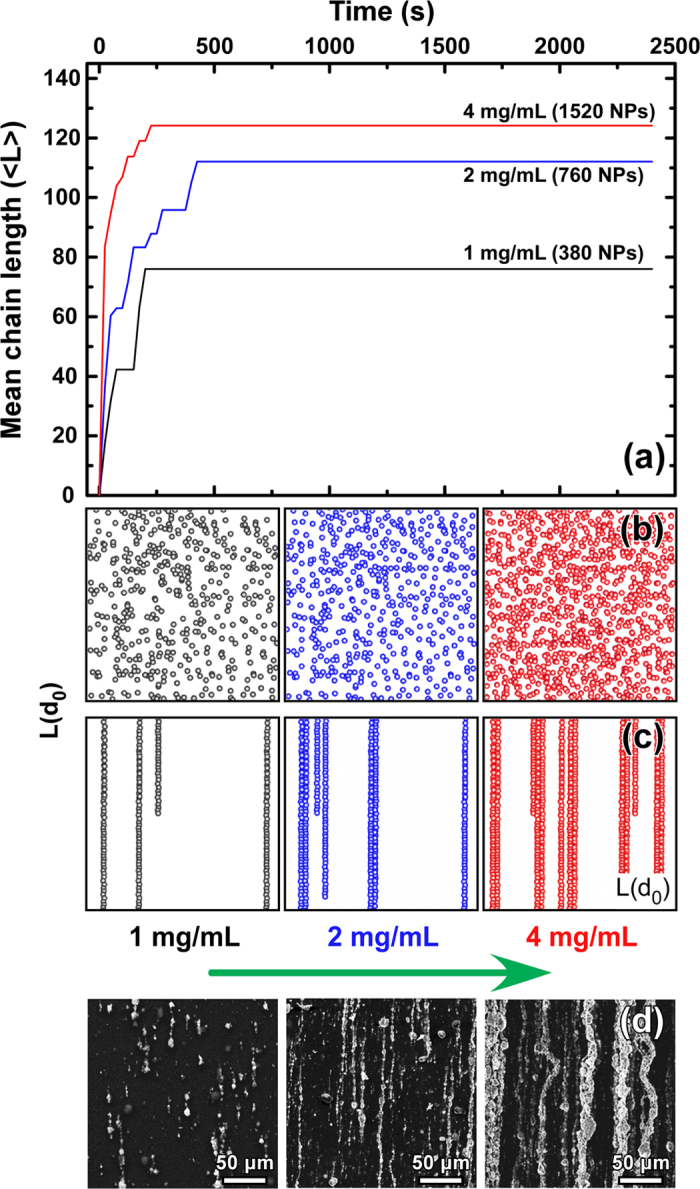
Molecular Dynamics simulations of chain formation of 40 nm MNPs for various concentration values. (**a**) Time evolution of the average length of the chains: as the simulation evolves, chains with increasing length appear. The length and density of chains increases with the particle concentration and is stabilized after 200 s (case of 1 and 4 mg/mL) and after 400 s (case of 2 mg/mL). (**b)** Projection in z-x plane of the randomly oriented MNPs in absence of external magnetic field. (**c**) Projection in z-x plane of the chain formation of MNPs in an external field of 40 mT. The dimensions of our 3D computational space were x = y = z = L(d_0_) = (80d_0_) where d_0_ is the MNPs diameter of 40 nm. The number of MNPs was set to 380, 760, 1520 for the concentrations of 1, 2 and 4 mg/mL, respectively. (**d**) Corresponding experimental SEM images for three MNPs concentrations.

The MD simulations revealed that the 40 nm nanoparticles assemble into linear chains parallel to the applied field (Fig. 4). The simulation starts from a pre-equilibrated system containing a specific number of particles which are randomly oriented (Fig. 4b, depicts the 2D projection for clarity reasons while the actual 3D space is presented in Fig. S4a). In absence of an external magnetic field, the particles do not arrange as magnetic dipolar chains. On the other hand, in the presence of a magnetic field, the magnetic nanoparticles acquire an effective magnetic dipole moment μ_s_ in the direction of the applied field, which induces the formation of linear chains of colloids (Fig. 4c, the actual 3D space is also presented in Fig. S4b). As the simulation evolves with time (Fig. 4a), MNPs driven by the field aggregate and chains with increasing width (number of adjacent nanoparticles) appear. The same behavior was observed for all concentration values: For higher concentrations longer and wider chains appear (Fig. 4a,c). In order to define the strength of interactions, the model assumes that all events, in which a nanoparticle enters into the interaction region of a chain, will lead to instantaneous aggregation^27^. At each time step *Δt* a random displacement in each direction is generated with a Gaussian distribution with zero mean and variance *2D*_*s*_*Δt* where *D*_*s*_ is the diffusion coefficient of the object (single particle or chain) in the direction of motion (*x*, *y*, *z*). Also during each time step the distances between objects are checked in order to detect penetration of an object inside the region of aggregation of another object or to detect possible overlaps between them. In Fig. 4a, each time snapshot (between two adjacent time steps) corresponds to a quasi-static condition during which no MNPs enter into the interaction region of individual chains where stronger interactions between nanoparticles take place. Moreover, as shown at Fig. 4c, the spacing between the chains can be effectively tuned, since it decreases with concentration. Results regarding the successful formation of chains, as well as information about the length and the inter-chains distances, qualitatively agree with SEM findings (Fig. 4d).

On the contrary, the 10 nm MNPs assembled into aggregates only with a minor tendency for chain formation (Fig. S3) due to the presence of much weaker magnetic inter-particle dipolar interactions. This is due to the fact that, for small particles, the thermal energy *k*_*B*_*T* is much higher than the magnetic interaction energy whereas for bigger particles the magnetic term prevails^23^,^27^.

**Figure 5:**
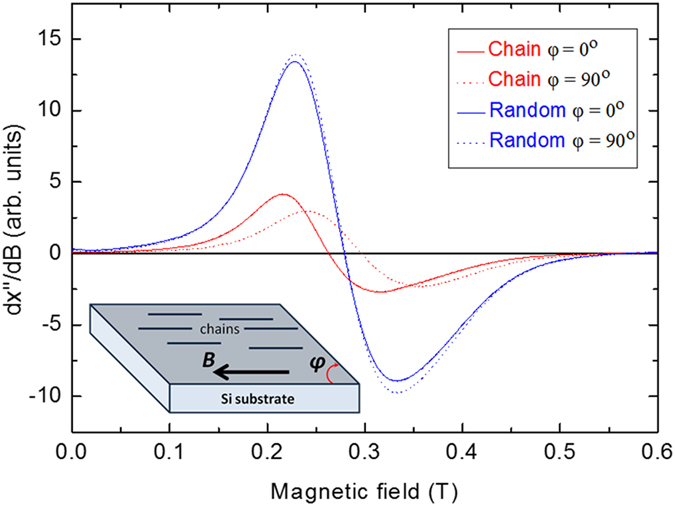
FMR spectra of random (blue lines) and chain (red lines) samples recorded at two orthogonal configurations of the applied magnetic field with respect to the sample’s alignment direction. As schematically shown in the inset, one configuration corresponds to φ = 0° (solid lines), where the field is parallel to the chain axis (arbitrary for the random sample), and a second configuration refers to φ = 90) (dashed lines), where the field is perpendicular to the chain axis. The spectra were taken for chain and random samples with 4 mg/mL concentration of 40 nm MNPs and 1 mg/mL of agarose content.

The dipolar coupling between magnetic nanoparticles in a chain induces magnetic shape anisotropy, which depends on inter-particle distance (filling factor) within a chain and on the chain aspect ratio^28^. The shape anisotropy contribution as the result of particles MNPs alignment (aggregation in a chain) can be revealed by FMR experiments as a shift of the resonance when obtained in parallel (magnetic easy axis) and perpendicular (magnetic hard axis) to the long axis of chains^29^. The FMR spectra for random and chain samples of 40 nm MNPs are shown in Fig. 5 with the magnetic field applied parallel and perpendicular to the samples’ alignment direction. For the randomly distributed MNPs (random sample), FMR spectra (blue lines) do not show any angular dependence, i.e., they do not present any shift of the FMR spectrum. For particles aligned in chains (chain sample), however, the resonance field (red lines) has a maximum when the magnetic field is applied perpendicular to the chain axis (φ = 90°) and minimum at parallel configuration (φ = 0°).

This angular dependence of the FMR line for the chain samples points towards the presence of uniaxial magnetic anisotropy with its axis parallel to the chain axis. A representative case of FMR spectra for the denser sample (4 mg/mL) comprised of 40 nm FM MNPs is given in Fig. 5 both for random and chain samples, while the complete series of FMR spectra for all samples and concentrations is presented in Fig. S5.

The distinction between the FMR spectra is depicted on the splitting of the resonance field when comparing random and chain samples. This indicates that the magnetic shape anisotropy is different for different chain samples due to possible variation of filling factors or aspect ratios of the chains. Our FMR measurements confirm the regular formation of magnetic chains as visualized in the SEM images (Figs 3 and 4d, and Fig. S3) and its effect on hyperthermia efficiency is discussed in the corresponding section.

**Figure 6:**
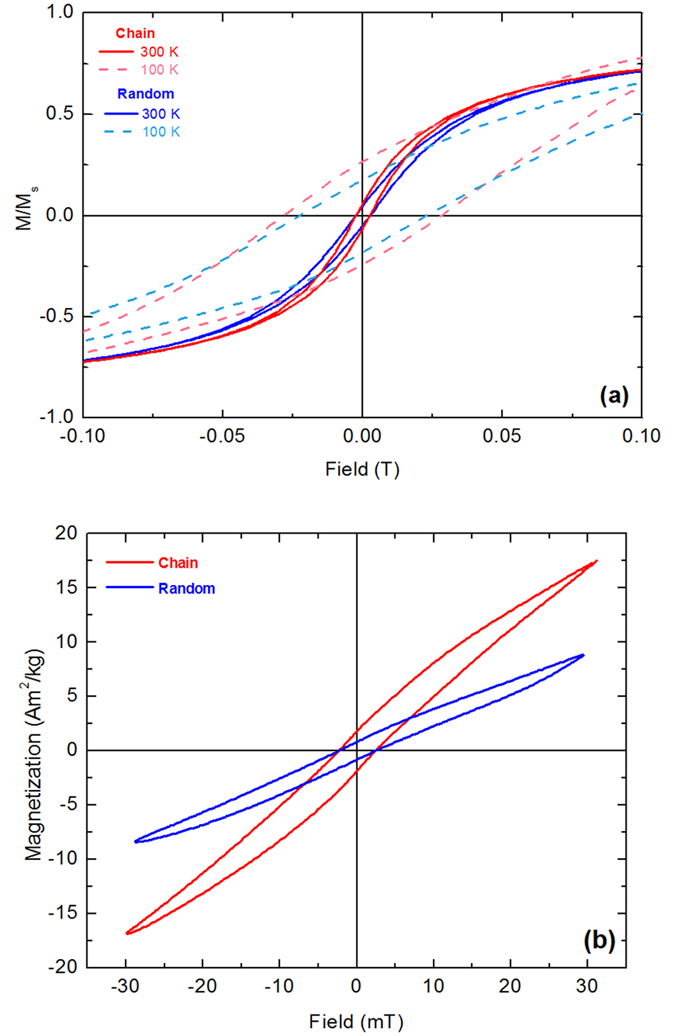
(**a**) Major magnetic hysteresis loops at 1 T of 40 nm MNPs at 100 and 300 K with the magnetic field applied parallel to the alignment direction of random (blue lines) and the chain (red lines) samples at 100 and 300 K. (**b**) Minor hysteresis loops of random (blue line) and chain (red line) samples recorded at 30 mT at 300 K.

Static hysteresis loops were recorded at 1 T for the chain and random 40 nm samples at 1 mg/mL agarose content and 4 mg/mL MNPs concentration at 100 and 300 K as shown in Fig. 6a, with the field applied parallel to the alignment direction of the chain sample. In the case of the chain sample, the faster magnetization rise compared to the random case, indicating that the susceptibility is larger^30^,^31^. Furthermore, at T = 100 K, the coercive field increases to 285 Oe for chains from 228 Oe for the random sample. Such a feature signifies the shape anisotropy induced by the chain formation, resulting to enhanced collective magnetic response and, thus, potentially to increased heating efficiency^32^.

Additionally, minor hysteresis loops were recorded at 30 mT (Fig. 6b) for both chain and random samples. As it can be observed in Fig. 6b, the hysteresis loop area of the chain sample (red loop) is larger than the random one (blue loop) which indicates that the energy density dissipated by the MNPs in chain sample is higher than the one of the random case. Based on the quasi-static magnetic behavior in 30 mT, i.e. the MPH magnetic field amplitude, we may evaluate a qualitative threshold of heat energy dissipation based on hysteresis loop area, keeping in mind that hysteresis loops increase in area under the dynamic conditions of MPH (kHz range).

Typical hyperthermia curves including heating and cooling cycles were recorded for all 10 nm and 40 nm samples with varying MNPs concentration and agarose content (the complete set of MPH is displayed in Fig. S6 and Fig. S7). There is a direct impact of collective magnetic features on heating efficiency, with MNPs covering a complete range of features from superparamagnetism at one end and ferromagnetism at the other^24^,^25^,^26^. In order to validate our experimental findings for the successfully formed chains of 40 nm MNPs, we employed the heat transfer module in COMSOL Multiphysics 3.5a^33^. The simulation is useful for understanding the basic features between the chains and random nanoparticles in a viscous liquid. Figure S8 shows that the experimental hyperthermia curves of chains show improved heating efficiencies when compared to random samples in excellent agreement with the corresponding simulations. The initial slope (directly affecting the SLP magnitude) of the chain sample is steeper as compared to the random reference sample. The simulation is performed by using the extended version of the classical model of Stoner and Wohlfarth for magnetic hysteresis accounting the temperature and frequency dependence of the coercive field^34^, we are able to estimate the hysteresis losses and reproduce the corresponding hyperthermia curves *T(t).*

The fundamental law governing all heat transfer is the first law of thermodynamics, commonly referred to as the principle of conservation of energy. However, internal energy, *U*, is a rather inconvenient quantity to measure and use in simulations. Therefore, the basic law is usually rewritten in terms of temperature, *T*. Assuming that mass is always conserved and the heat transfer interfaces use Fourier’s law of heat conduction, which states that the conductive heat flux, *Q*, is proportional to the temperature gradient, the equation governing pure conductive heat transfer between interfaces if the velocity is set to zero, is obtained:


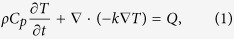


where *ρ* is the density (kg/m^3^), *C*_*p*_ is the specific heat capacity at constant pressure (J/(kg·K)), *T* is absolute temperature (K) and *Q* contains heat sources other than viscous heating (W/m^3^). The heat source, given by the hysteresis losses, is set to our simulations equal to


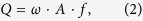


where *A* is the hysteresis loop area *f* is the frequency and *ω* is the MNPs volume fraction which is equal to the MNPs concentration divided to their density and represents the fraction of nanoparticles in the solution. Specifically, for “hysteretic” MNPs where stronger magnetic interactions take place, power losses are attributed to the hysteresis losses^35^,^36^ with the following analytical expression for hysteresis loop area:


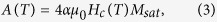


where *A* is the area enclosed by the hysteresis loop representing the amount of MNPs energy loss when the latter are subjected to an external magnetic field and *α* is a coefficient including the shape deviation from a perfectly square hysteresis loop and is equal to 0.5 for random oriented and 0.75 for aligned MNPs. The saturated magnetization M_sat_ was taken by the minor hysteresis loop (Fig. 6b) and is equal to 18 Am^2^/kg for the chain sample and 8 Am^2^/kg for the random sample, while the coercive field is estimated from an extended Stoner-Wohlfarth model. (See details for theoretical estimations at Supporting Information).

**Figure 7:**
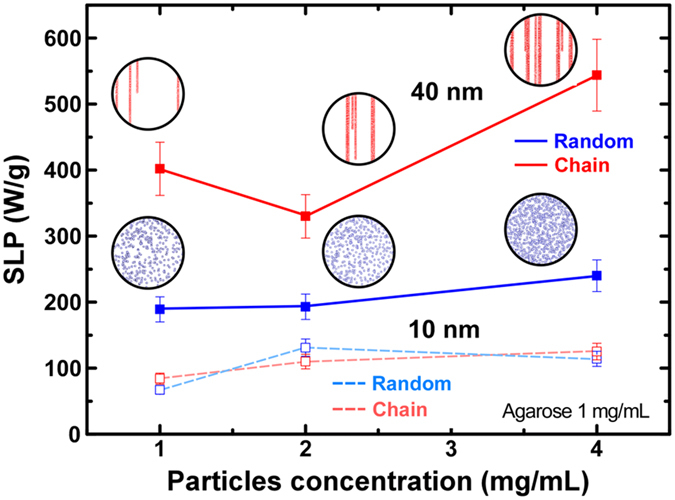
MNPs’ alignment influence on magnetic hyperthermia efficiency as expressed by SLP values by varying MNPs’ concentrations for two different configurations (random and chain) at 765 kHz frequency and 30 mT field with MNPs sizes of 40 nm (solid symbol) and 10 nm (open symbol). For chain samples, SLP values refer to measurements performed with the AC hyperthermia field applied parallel to the alignment direction of the chain samples.

Additional degrees of freedom for successful chain formation and eventual superior heating performance may be provided by taking into account pH variations and medium’s viscosity. Parameters affecting the dipolar interaction are both the intrinsic magnetic dipole moment and their inter-particle distances. Intrinsic magnetic moments are governed mainly by MNPs size resulting in our case either to SPM (10 nm) or to FM (40 nm) MNPs. Inter-particle distances may be tuned via MNPs concentration and agarose content. In Fig. 7 the influence of the parameters affecting dipolar interaction intensity on the heating efficiency is depicted. Figure 7 presents the MNPs concentration dependence (1–4 mg/mL) of SLP for 10 nm and 40 nm MNPs in the case of random samples and the corresponding dependence of chain samples at constant agarose content of 1 mg/mL and under AC field of 765 kHz and 30 mT. From Fig. 7 (open symbols) it seems that SLP values for 10 nm do not present significant differences between the random and the chain samples. This may be ascribed to their intrinsic superparamagnetic response (in quasi-static VSM measurements), resulting to milder influence of external magnetic field chain formation effects. However, it should be noted that, in high frequency heating experiments, the nanoparticle systems of 10 nm may also exhibit dissipation since wider hysteresis loops arise on the time scale of the experiment, yet such an effect remains small since it initiates practically from SPM particles. As a matter of fact, we assume that the thinner and shorter chains formed in the 10 nm MNPs aligned samples do not eventually manage to enhance SLP values. In such a case, a greater, in magnitude, alignment magnetic field might lead to the formation of thicker and longer chains even and resolve SLP increase issues.

On the other hand for the 40 nm MNPs chain samples (Fig. 7 solid symbols), there is a systematic enhancement of heating efficiency, as illustrated by the SLP values, compared to corresponding values of random samples, regardless of concentration. It is clear that in the 40 nm MNPs the SLP values are almost two times larger for the chain samples as compared to their random counterpart. Accordingly, in agreement with the MD simulations, SEM images and FMR measurements, we may surmise that the larger the MNPs’ concentration, the longer and wider were the chains and this is readily reflected to the SLP values.

In an effort to examine the role of medium’s viscosity to the chain formation and thermal response, we have measured the heating efficiency of a series of samples (random and chain) with varying agarose content, while MNPs concentration was kept constant at 4 mg/mL. Figure S9 and S10 present the SLP values with agarose content from 0.5 to 20 mg/mL for the 40 nm and 10 nm MNPs respectively random and chain samples. One can see that, starting from 1 mg/mL agarose content, different SLP values appear for random and chain samples. By increasing agar content, in other words, solution viscosity, SLP values decrease for both samples. Solutions of agarose content ≥5 mg/mL, possess viscosity values that practically attenuate dipolar interactions and consequent chain formation. However, in any case aligned chain samples present larger values than random samples. Similar behavior was also observed at the lower frequency of 210 kHz (supplementary section Fig. S9b). As expected, this effect is practically not observed, for the 10 nm SPM MNPs, even at the high frequency (765 kHz) field (supplementary Fig. S10) due to the formation of thinner and shorter chains.

## Conclusions

The role of chain formation to further optimize the heating efficiency of magnetic particle hyperthermia was studied for 10 and 40 nm magnetically aligned Fe_3_O_4_ MNPs. Thicker, denser and longer chains could be successfully formatted in the case of the 40 nm MNPs as compared to their 10 nm ones, due to the presence of stronger dipolar interactions. Chain formation induces shape anisotropy and results to magnetic heating efficiency enhanced by up to a factor of two for the denser 4 mg/mL, 40 nm MNPs chain sample. Molecular Dynamics anisotropic diffusion model used to simulate the chain formation process results to a very good agreement with experimental SEM images. The chains’ width, length and density depend not only on MNPs size but on colloidal parameters of the agarose solution as well. A modified Stoner Wohlfarth model was used to successfully simulate experimental heating curve hyperthermia measurements. Critical parameters, such as size, alignment field intensity, colloidal parameters and intrinsic magnetic properties such as magnetization, may be fine-tuned to optimize the heating efficiency of aligned MNPs. Consequently, as an alternative to conventional material selection schemes, the pathway in the magnetic particle hyperthermia material roadmap widens just by arranging the ‘proper’ particles at the nanoscale.

## Methods

### Synthesis of particles

Magnetite nanoparticles were prepared by the aqueous co-precipitation of ferric and ferrous salts at alkaline conditions at high temperature^37^. To receive superparamagnetic Fe_3_O_4_ MNPs (average diameter about 10 nm), FeCl_2_ and FeCl_3_ were dissolved in distilled water and heated up to 80 °C under continuous stirring. At this point, a quantity of 0.1 M NaOH solution was rapidly added to obtain a pH of 10.5 and a black dispersion, indicating the formation of nanoparticles. After 2 h ageing by slow stirring, the dispersion was cooled down to ambient temperature followed by a sequence of washing and centrifuging cycles to obtain particles in dried form or as a water dispersion. The replacement of iron reagents by Fe_2_(SO_4_)_3_ and (NH_4_)_2_Fe(SO_4_)_2_ modifies the nucleation process resulting in a significant size increase from 10 to 40 nm Fe_3_O_4_ MNPs. i.e. single-domain ferromagnetic particles at ambient temperature. In both cases, the total concentration of iron reagents was 0.5 M and the Fe^2+^ to Fe^3+^ ratio was equal to the Fe_3_O_4_ stoichiometry (1:2).

### Chain formation in agarose

Chain formation at varying content gel matrix followed the MNPs synthesis. In each case, the designated mixture of magnetite in different concentrations (10 or 40 nm) MNPs (1, 2, 4 mg/mL) and different agarose content (0.5, 1, 2, 5, 10, 15, 20 mg/mL) was prepared in 1 mL of deionized water (two identical samples), homogenized with sonication and magnetic stirring in a water bath of 84 °C. Finally, one solution sample was subjected to a static magnetic field of 40 mT for 40 minutes (see the Supporting Information Figure S1a for details) to induce the chain formation of the nanoparticles (chain sample) while a second solution sample was left to cool down in the absence of a magnetic field, yielding the reference sample with random nanoparticle dispersion (random sample).

### Structure and morphology

The structure of the MNPs was identified by X-ray powder diffraction (XRD) using a two-cycle Rigaku Ultima + X-ray diffractometer with a CuK_α_ radiation operating at 40 kV/30 mA. We set the two theta step to 0.05° and accumulated the signal for 3 s in Bragg-Brentano geometry. The chain formation of MNPs in agarose was investigated by scanning electron microscopy (SEM) using Zeiss LEO 1530 SEM on Si substrates. Prior to SEM, the specimens were exposed to a mild reactive oxygen plasma for partial removal of the agarose. Such a treatment accounts for the surface imaging in SEM and does not affect the lateral distribution of MNP chains or the number of Fe atoms per particle^38^.

### Magnetic characterization

Magnetic hysteresis loops were recorded at 100 and 300 K using an Oxford Instruments 1.2 H/CF/HT VSM. Zero field cooling/field cooling (ZFC/FC) experiments were performed in a temperature range of 10 to 300 K at an applied field of 5 mT using a Quantum Design Dynacool PPMS system. Ferromagnetic resonance (FMR) measurements were obtained using a conventional Bruker X-band spectrometer (ELEXSYS-II E580) which operated at microwave frequency of 9.5 GHz. The FMR spectra were recorded by sweeping the magnetic field (*B*) as applied parallel to the Si substrate surface in two different configurations: parallel (*φ* = 0°) and perpendicular (*φ* = 90°) with respect to the alignment direction of the chain samples (the chain axis) as it is schematically shown in the inset of Fig. 5.

### Magnetic Particle Hyperthermia

Magnetic particle hyperthermia experiments were performed using a 1.2 kW Ambrell Easyheat 0112 operating at 210 kHz and a 4.5 kW inductive heater operating at 765 kHz both under an AC induction field amplitude of 30 mT. Each measurement cycle included a heating and a cooling step. The temperature was continuously recorded (0.4 s steps) using a GaAs-based fiber optic probe immersed in a vial containing 1 mL of solution with the AC hyperthermia field applied parallel to the alignment direction of the chain samples. The heating efficiency of MNPs is quantified by the specific loss power values (SLP) determined from the power absorption per unit mass of magnetic material (in W/g) following a standardized procedure to solely estimate the magnetic heating contribution^39^,^40^, by using equation:


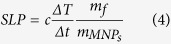


where *c* is the specific heat capacity of the sample, m_f_ the ferrofluid dispersion mass, *m*_*MNPs*_ is the iron oxide mass diluted in the dispersion and *ΔΤ/Δt* is the fitted initial temperature gradient after inductive heating started.

### Computational procedures

Two distinct computational procedures were implemented as follows:

1. **Molecular Dynamics Algorithm:** In order to gain a better understanding about the driving force of chain formation, we conducted Molecular Dynamics (MD) simulations by using the MagChain package^27^. This approach consists of two key parts: a) the first one is the diffusion model adopted to describe the motion of the coarse-grain (CG) objects following the Brownian dynamics, where a chain containing s >1 particles exhibits anisotropic diffusion. The diffusion coefficient for a single, isolated MNP is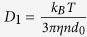, where *η* is the viscosity of the fluid. A chain containing at least two nanoparticles exhibits anisotropic diffusion, characterized by a diffusion coefficient 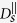 in the direction parallel to the long axis of the chain and 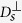 in the direction perpendicular to the long axis. In order to define those coefficients we used the following expressions adopted from Ref. 41 (slender body theory): 
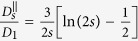
 and 
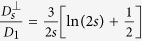
 where *s* is the length of the chain (number of MNPs). b) The second one is the dipolar interaction strength between MNPs with respect to the thermal energy expressed by the magnetic coupling parameter 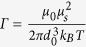 where *μ*_*s*_ is the magnetic dipole moment of the individual MNP at saturation. Each CG object is surrounded by an aggregate region in which the magnetic interaction between the CG object and a dummy single particle takes place. This region depends on the size of the considered aggregate and on the magnetic coupling parameter. For small chains the attraction radius increases with their size and tends to a constant value for larger chains. The aggregate region is described by a spherical attraction zone at the poles of each MNP or aggregate. These zones are defined by the radius *r*_*a*_ given by the solution of equation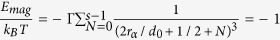. The values of *r*_*a*_*(s)* are calculated by finding the distance at which the magnetic interaction energy *E*_*mag*_ between a chain of s particles and a single test particle is equal to *−k*_*B*_*T* (at the edge of the spherical attraction zone). In the case of aggregation (i.e. when the MNPs are within the zone) of two nanoparticles we set 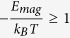, and a new “extended” nano-entity is created. In the case of aggregation of two CG objects, a new CG object is created (and the two previous CG objects are erased from the simulation) with length s, obtained from adding the lengths of the two aggregating chains and located at the center of mass of the aggregating CG objects. This was the case for the 40 nm MNPs while for the 10 nm MNPs we found that the above condition for nanoentity formation is not satisfied since the thermal energy was much higher than the magnetic one: *E*_*mag*_/*k*_*B*_*T* ≪ 1. As a result, the 10 nm system is not able to form linear chains but it forms aggregates due to weak dipolar interactions. More details for this process appear in Supporting Information.
2. **Stoner-Wohlfarth model based theory for hysteresis loop estimation:** With a modified Stoner-Wohlfarth model based theory for hysteresis loop estimation we may quantify the heating properties of MNPs systems which are far from the linear response regime incorporating the role of finite temperature and frequency on the coercive field. The ultimate outcome of this approach is the prediction of the specific loss power values and their direct comparison with experimental ones. Thus, we may unravel the decisive role of distinct collective magnetic features and consequent magnetic tuning via dipolar interactions on magnetic heating efficiency. Details for this procedure appear in Supporting Information section.

## Additional Information

**How to cite this article**: Myrovali, E. *et al*. Arrangement at the nanoscale: Effect on magnetic particle hyperthermia. *Sci. Rep.* **6**, 37934; doi: 10.1038/srep37934 (2016).

**Publisher's note:** Springer Nature remains neutral with regard to jurisdictional claims in published maps and institutional affiliations.

## Supplementary Material

Supplementary Information

## References

[b1] Dutz S. & Hergt R. Magnetic particle hyperthermia–a promising tumour therapy? Nanotechnology 25, 452001 (2014). 25337919 10.1088/0957-4484/25/45/452001

[b2] Périgo E. A. . Fundamentals and advances in magnetic hyperthermia. Applied Physics Reviews 2, 041302 (2015).

[b3] Martinez-Boubeta C. . Self-assembled multifunctional Fe/MgO nanospheres for magnetic resonance imaging and hyperthermia. Nanomedicine: Nanotechnology, Biology, and Medicine 6, 362–370 (2010). 10.1016/j.nano.2009.09.003 19800988

[b4] Martinez-Boubeta C. . Learning from nature to improve the heat generation of iron-oxide nanoparticles for magnetic hyperthermia applications. Scientific reports 3, 1652 (2013). 23576006 10.1038/srep01652 PMC3622918

[b5] De Montferrand C. . Iron oxide nanoparticles with sizes, shapes and compositions resulting in different magnetization signatures as potential labels for multiparametric detection. Acta Biomaterialia 9, 6150–6157 (2013). 23207434 10.1016/j.actbio.2012.11.025

[b6] Tripp S. L., Pusztay S. V., Ribbe A. E. & Wei A. Flux Closure in Self-Assembled Cobalt Nanoparticle Rings. Journal of the American Chemical Society 124, 7914–7915 (2002). 12095331 10.1021/ja0263285

[b7] Spasova M. . Magnetic Properties of Arrays of Interacting Co Nanocrystals. J. Mag. Mag. Mater. 240, 40–43 (2002).

[b8] Fragouli D. . Dynamical formation of spatially localized arrays of aligned nanowires in plastic films with magnetic anisotropy. ACS Nano 4, 1873–1878 (2010). 20356062 10.1021/nn901597a

[b9] Fragouli D. . Formation and microscopic investigation of iron oxide aligned nanowires into polymeric nanocomposite films. Microscopy research and technique 73, 952–8 (2010). 20232460 10.1002/jemt.20848

[b10] Cozzoli P. D. . Colloidal synthesis and characterization of tetrapod-shaped magnetic nanocrystals. Nano letters 6, 1966–1972 (2006). 16968009 10.1021/nl061112c

[b11] Conde-Leborán I., Serantes D. & Baldomir D. Orientation of the magnetization easy axes of interacting nanoparticles: Influence on the hyperthermia properties. Journal of Magnetism and Magnetic Materials 380, 321–324 (2015).

[b12] Ilg P. & Kröger M. Anisotropic self-diffusion in ferrofluids studied via Brownian dynamics simulations. Physical Review E - Statistical, Nonlinear, and Soft Matter Physics 72, 031504 (2005). 10.1103/PhysRevE.72.031504 16241441

[b13] Wang A., Li J. & Gao R. The structural force arising from magnetic interactions in polydisperse ferrofluids. Applied Physics Letters 94, 212501 (2009).

[b14] Li H., Peng X. & Chen W. Simulation of the Chain-formation Process in Magnetic Fields. Journal of Intelligent Material Systems and Structures 16, 653–658 (2005).

[b15] Bizdoaca E. L. . Magnetically directed self-assembly of submicron spheres with a Fe_3_O_4_ nanoparticle shell. Journal of Magnetism and Magnetic Materials 240, 44–46 (2002).

[b16] Salgueiriño-Maceira V. . One-dimensional assemblies of silica-coated cobalt nanoparticles: Magnetic pearl necklaces. Journal of Magnetism and Magnetic Materials 303, 163–166 (2006).

[b17] Serantes D. . Multiplying Magnetic Hyperthermia Response by Nanoparticle Assembling. The Journal of Physical Chemistry C 118, 5927–5934 (2014).

[b18] Toulemon D. . Enhanced Collective Magnetic Properties Induced by the Controlled Assembly of Iron Oxide Nanoparticles in Chains. Advanced Functional Materials. 26, 2454–2462 (2016).

[b19] Prozorov R. . Magnetic irreversibility and the Verwey transition in nanocrystalline bacterial magnetite. Physical Review B - Condensed Matter and Materials Physics 76 (2007).

[b20] Fernández E. . Rheological and thermal properties of agarose aqueous solutions and hydrogels. Journal of Polymer Science, Part B: Polymer Physics 46, 322–328 (2008).

[b21] Chen Z. J., Broaddus W. C., Viswanathan R. R., Raghavan R. & Gillies G. T. Intraparenchymal drug delivery via positive-pressure infusion: Experimental and modeling studies of poroelasticity in brain phantom gels. IEEE Transactions on Biomedical Engineering 49, 85–96 (2002). 12066887 10.1109/10.979348

[b22] Salloum M., Ma R. H., Weeks D. & Zhu L. Controlling nanoparticle delivery in magnetic nanoparticle hyperthermia for cancer treatment: experimental study in agarose gel. International journal of hyperthermia: the official journal of European Society for Hyperthermic Oncology, North American Hyperthermia Group 24, 337–345 (2008). 10.1080/02656730801907937 18465418

[b23] Bertoni G. . Nanochains formation of superparamagnetic nanoparticles. Journal of Physical Chemistry C 115, 7249–7254 (2011).

[b24] Cacciola M. & Osaci M. Studies about the Influence of Self-Organization of Colloidal Magnetic Nanoparticles on the Magnetic Néel Relaxation Time. Colloid Journal 78, 4, 448–458 (2016).

[b25] Branquinho L.C. . Effect of magnetic dipolar interactions on nanoparticle heating efficiency: Implications for cancer hyperthermia. Scientific Reports 3, 2887 (2013). 24096272 10.1038/srep02887 PMC3791447

[b26] Ruta S., Chantrell R. & Hovorka O. Unified model of hyperthermia via hysteresis heating in systems of interacting magnetic nanoparticles. Scientific Reports 5, 9090 (2015). 25766365 10.1038/srep09090 PMC5155484

[b27] Andreu J. S., Calero C., Camacho J. & Faraudo J. On-the-fly coarse-graining methodology for the simulation of coupled field-induced aggregation and sedimentation processes arising chain formation of superparamagnetic colloids in strong magnetic fluids. Physical Review E 85, 036709 (2012). 10.1103/PhysRevE.85.036709 22587211

[b28] Wiedwald U., Spasova M., Farle M., Hilgendorff M. & Giersig M. Ferromagnetic resonance of monodisperse Co particles. Journal of Vacuum Science & Technology A: Vacuum, Surfaces, and Films 19, 1773 (2001).

[b29] Liébana-Viñas S. . Magnetic hardening of Fe_30_Co_70_ nanowires. Nanotechnology 26, 415704 (2015). 26404670 10.1088/0957-4484/26/41/415704

[b30] Nakata K., Hu Y., Uzun O., Bakr O. & Stellacci F. Chains of superparamagnetic nanoparticles. Advanced Materials 20, 4294–4299 (2008).

[b31] Gross A. F., Diehl M. R., Beverly K. C., Richman E. K. & Tolbert S. H. Controlling Magnetic Coupling between Cobalt Nanoparticles through Nanoscale Confinement in Hexagonal Mesoporous Silica. Journal of Physical Chemistry B 107, 5475–5482 (2003).

[b32] Barcikowski S., Baranowski T., Yigit Durmus Y., Wiedwald U. & Gökce B., Solid solution magnetic FeNi nanostrand–polymer composites by connecting-coarsening assembly. Journal of Materials Chemistry C 3, 10699–10704 (2015).

[b33] Comsol Multiphysics Tutorial, AC/DC Model & General Heat Model, version 3.5a.

[b34] Stoner E. C. & Wohlfarth E. P. A mechanism of magnetic hysteresis in heterogeneous alloys (reprinted from philosochical transaction royal society-London, Vol 240, Pg 599-642, 1948). IEEE Transactions on Magnetics 27, 3475–3518 (1991).

[b35] Carrey J., Mehdaoui B. & Respaud M. Simple models for dynamic hysteresis loop calculations of magnetic single-domain nanoparticles: Application to magnetic hyperthermia optimization. Journal of Applied Physics 109 (2011).

[b36] Mehdaoui B. . Increase of magnetic hyperthermia efficiency due to dipolar interactions in low-anisotropy magnetic nanoparticles: Theoretical and experimental results. Physical Review B - Condensed Matter and Materials Physics 87 (2013).

[b37] Simeonidis K. . A versatile large-scale and green process for synthesizing magnetic nanoparticles with tunable magnetic hyperthermia features. RSC Adv. 6, 53107 (2016).

[b38] Wiedwald U. . From colloidal Co/CoO core/shell nanoparticles to arrays of metallic nanomagnets: Surface modification and magnetic properties. Chem. Phys. Chem 6, 2522–2526 (2005). 16270368 10.1002/cphc.200500148

[b39] Chalkidou A. . *In vitro* application of Fe/MgO nanoparticles as magnetically mediated hyperthermia agents for cancer treatment. Journal of Magnetism and Magnetic Materials 323, 775–780 (2011).

[b40] Simeonidis K. . Fe-based nanoparticles as tunable magnetic particle hyperthermia agents. Journal of Applied Physics 114, 103904 (2013).

[b41] Happel J. & Brenner H. Low Reynolds number hydrodynamics. (Cambridge 1985).

