# Supplementary material for: Arrangement at the nanoscale: Effect on magnetic particle hyperthermia
Source: Sci Rep. 2016 Nov 29;6:37934. doi: 10.1038/srep37934 (PMC5126575; doi:10.1038/srep37934)
Supplement: Supplementary Information [file srep37934-s1.pdf]

Arrangement at the nanoscale: Effect on magnetic particle hyperthermia

E.Myrovali[1], N. Maniotis[1], A. Makridis[1], A. Terzopoulou[1], V. Ntomprougkidis[1], K. Simeonidis[1], D. Sakellari[1], O. Kalogirou[1], T. Samaras[1], R. Salikhov[2], M. Spasova[2], M. Farle[2], U. Wiedwald[2,*] and M. Angelakeris[1,*]

[1] Physics Department, Aristotle University of Thessaloniki, Thessaloniki, 54124, Greece
[2] Faculty of Physics and Center for Nanointegration Duisburg-Essen (CENIDE), University of Duisburg-Essen, 47048 Duisburg, Germany

** Corresponding authors: ulf.wiedwald@uni-due.de, agelaker@auth.gr*

Supplementary Information

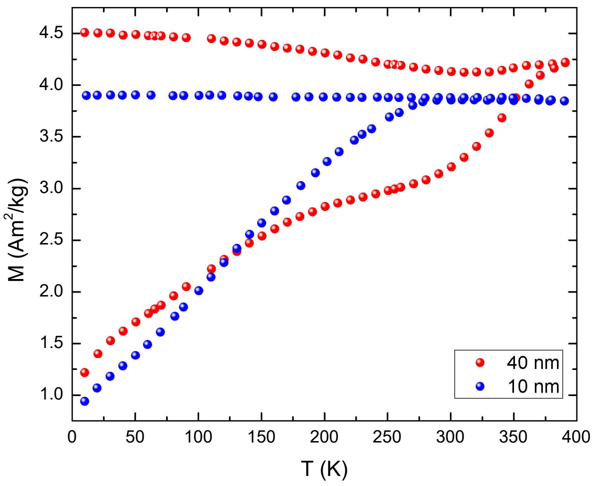

Figure S1: *ZFC/FC curves between 10 and 390 K for both powder MNPs samples recorded under 5 mT magnetic field.*

The preparation of magnetite colloidal agarose solutions with varying MNPs and agarose concentrations was carried out in a two-step process. At the first stage MNPs were synthesized using the co-precipitation method as described in [1]. The second stage starts with the solution dispersions. Different mixtures of magnetite (10 or 40 nm) MNPs' ferrofluids (1, 2, 4 mg/mL) and agarose solutions (0.5, 1, 2, 5, 10, 15, 20 mg/mL) were prepared in 1 mL of deionized water. Two identical samples from each batch were sonicated for 1 min and then placed in a water bath of 84°C under continuous magnetic stirring and sonication for three

minutes. Finally, the two solutions were allowed to cool down to room temperature to form the agarose gel. During this 40 minutes stage, one solution was subjected into a static magnetic field of 40 mT (Fig. S2a) to form the "chain sample". The second solution was left to cool down in the absence of magnetic field to serve as reference sample with random nanoparticle dispersion within its volume ("random sample").

In Figure S2a, the device used to align the nanoparticles is shown with two cubic NdFeB magnets located on top and bottom of the glass vial containing the colloidal sample. Using the COMSOL 3.5a packet we simulated the spatial arrangement, and we estimated the magnetic field distribution within the sample region (Fig. S2b). We have also calculated the magnetic flux density distribution in the magnetic orientation setup for 2D (Fig.S2c) and 3D mapping (Fig.S2d). In order to find the optimum parameters, for the externally applied magnetic field leading to chains' formation, we performed two experiments shown in Figs. S2e and S2f. Fig. S2e clearly outlines the relationship between the distance of sample-magnet and the magnetic field intensity. Preliminary results showed that the maximum heating efficiency (SLP ~480 W/g in the case of the 40 nm sample with MNPs concentration 4 mg/mL and agarose content 2 mg/mL) was achieved when the sample was subjected at a magnetic field of around 40 mT in order to achieve chains formation. Thus, we were able to determine the appropriate sample-magnet relative positions in order to get the maximum heating efficiency i.e. ~7 cm where a field of 40 mT is obtained. Cumulative MPH sequences performed on the same chain sample, resulted to reproducible SLP values. Thus, we concluded that the hyperthermia field of 30 mT used for the measurements, not only successfully penetrates the chain samples, but it does not affect their morphology.

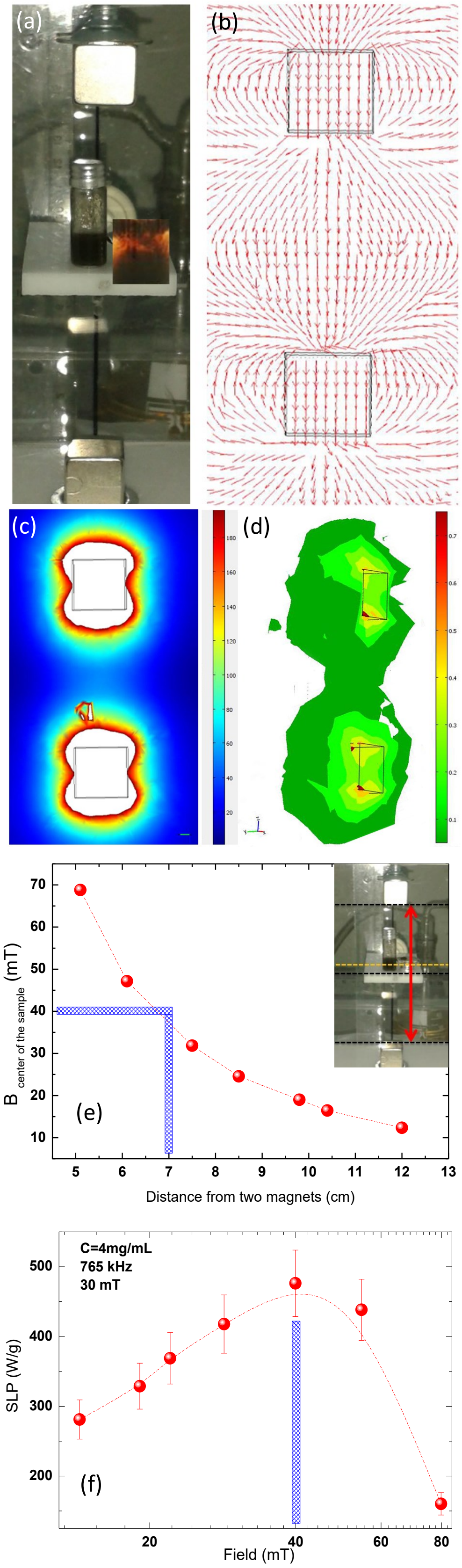

Figure S2: (*a) Experimental device for the chain formation. The sample is centered inside a homogeneous magnetic static field created by the two cubic NdFeB magnets, (b) Simulation of the magnetic field created from the two magnet-array (c) 2D calculated flux density distribution in the device region, (d) 3D mapping of the static magnetic field (e) magnetic field attenuation with respect to distance of poles from sample's center (f) Optimum magnetic field strength as dictated by maximizing heating efficiency (SLP).*

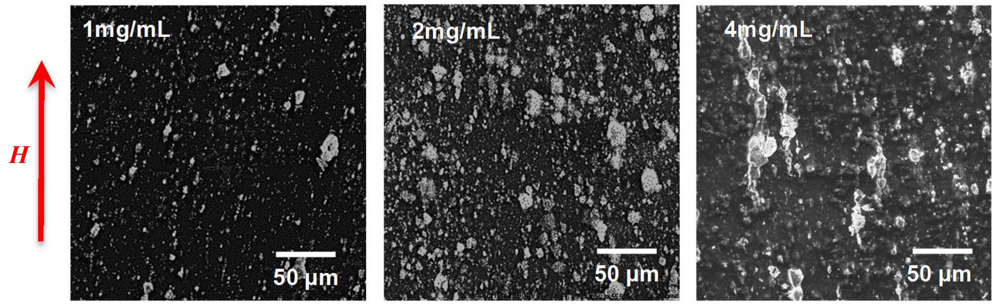

Figure S3: *SEM images for 10 nm MNPs formed in a magnetic field H of 40 mT with varying MNPs concentration (indicated in the images) and constant agarose content of 1mg/mL. External field direction is shown by the red arrow.*

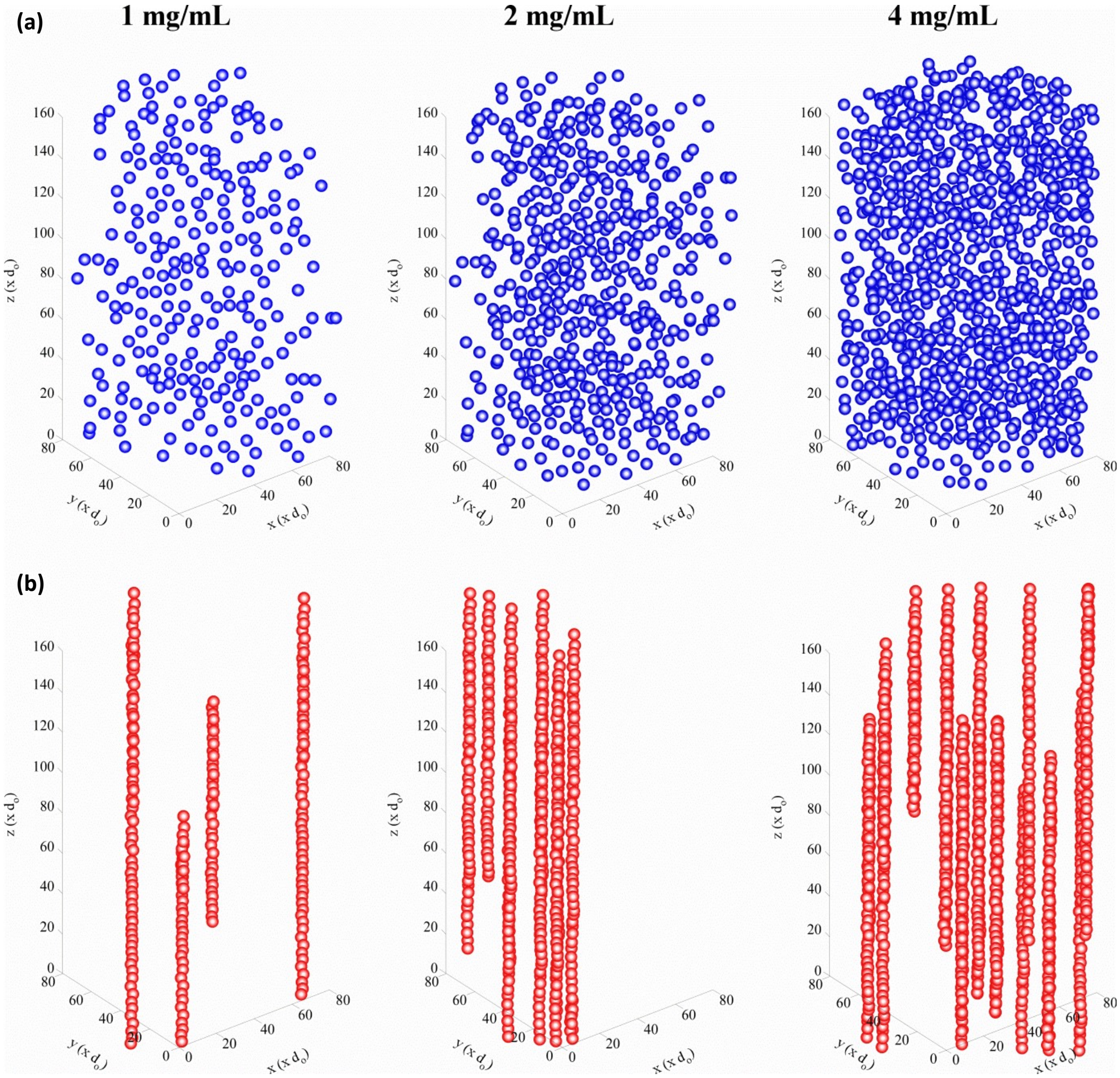

Figure S4: *(a) Randomly oriented MNPs in absence of the external magnetic field. (b) Chain formation of MNPs in an external field of 40 mT. The dimensions of our 3D computational space were $x=y=L(d_0)=(80d_0)$ and $z=(160d_0)$ where d_0 is the MNPs diameter of 40 nm. The number of MNPs was set to 380, 760, 1520 used for the concentrations of 1, 2 and 4 mg/mL, respectively.*

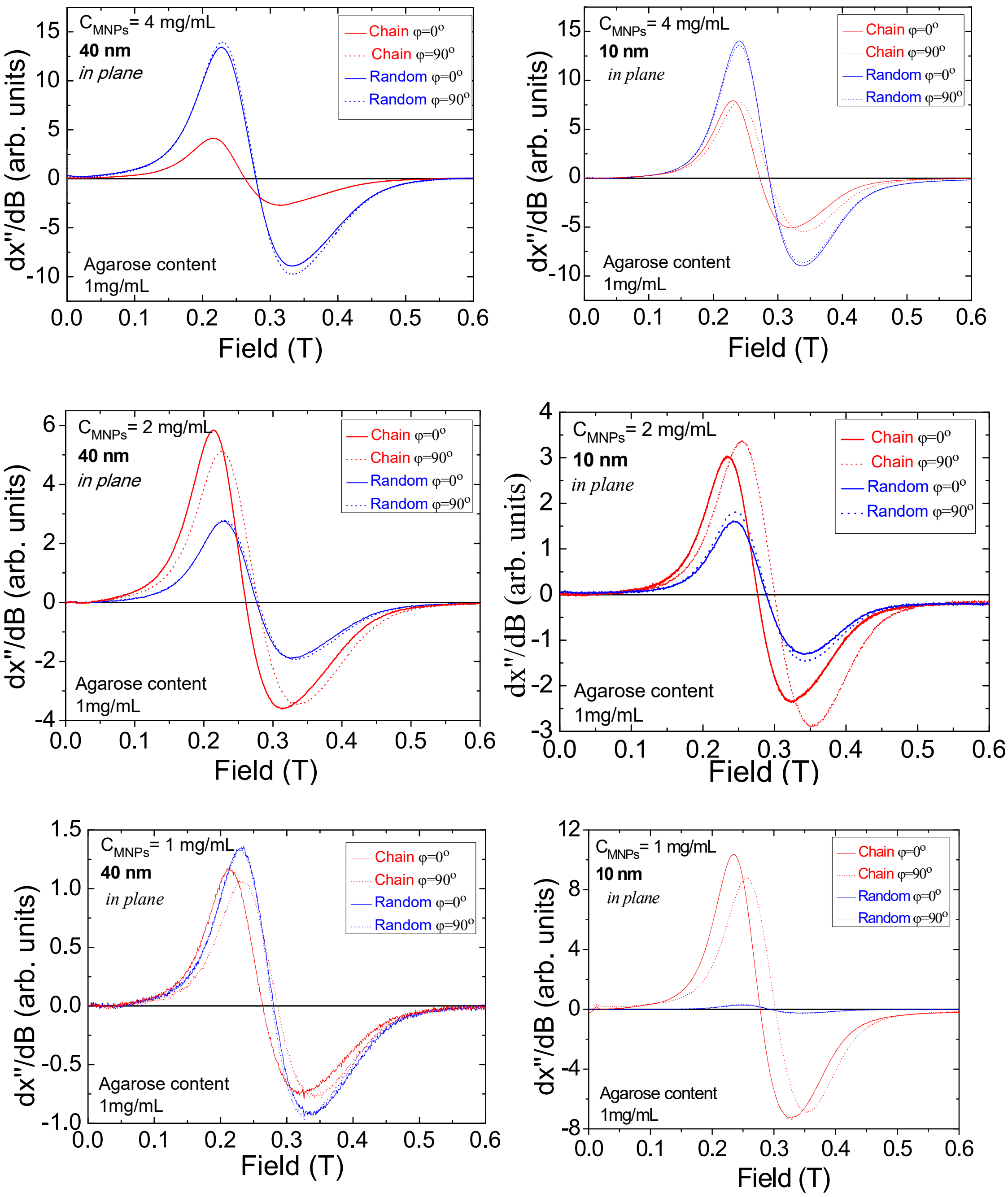

Figure S5: *FMR spectra of random and chain samples recorded at room temperature and at 9.5 GHz with the field applied parallel (φ=0º) and perpendicular (φ=90º) to the samples' alignment direction with respect to the chain samples for three different MNPs concentrations 1, 2 and 4 mg/mL, respectively.*

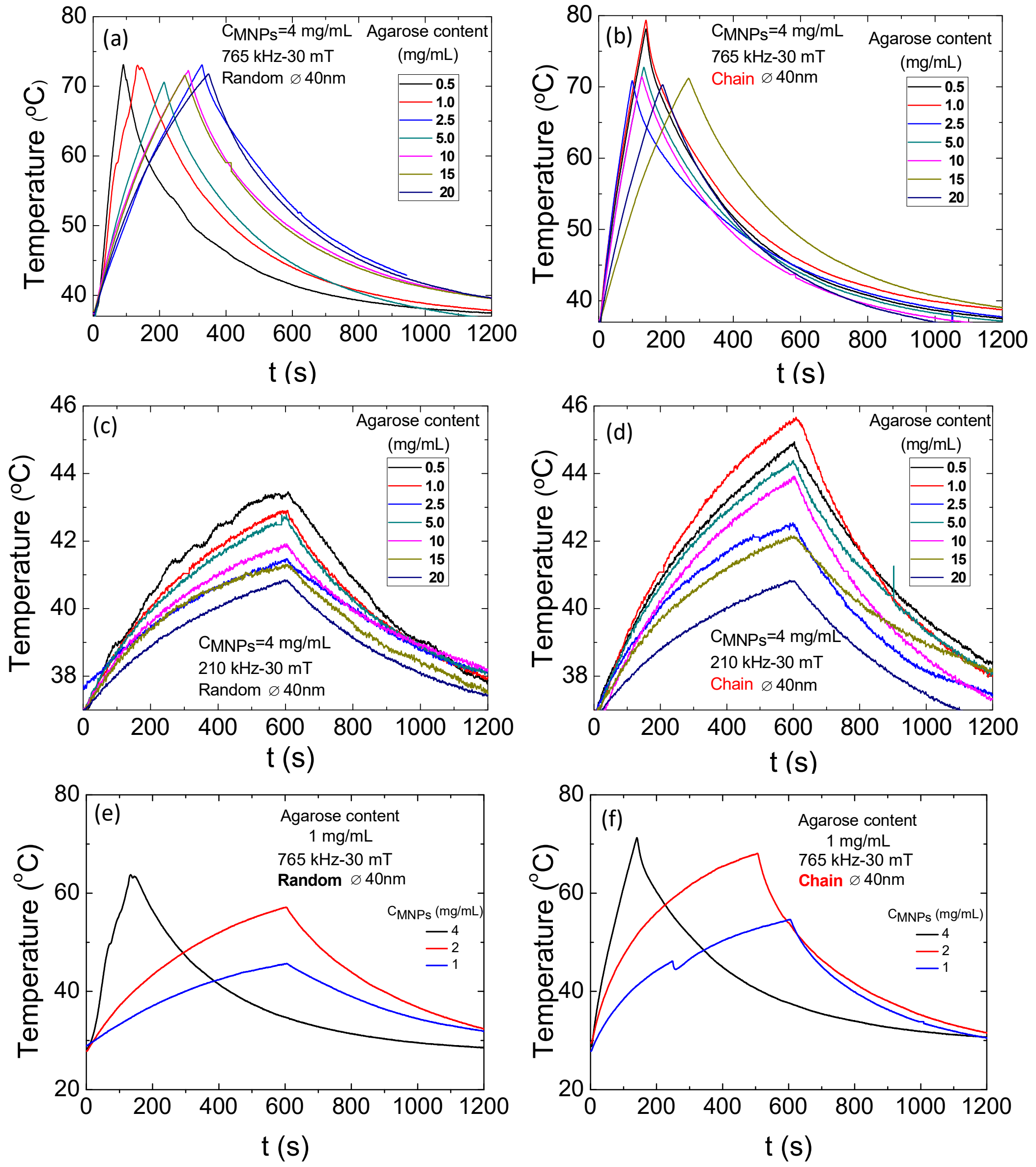

Figure S6*: Magnetic Particle Hyperthermia: Time-dependent temperature curves of 40nm random and chain samples for varying agarose content and 4mg/mL MNPs concentration under AC field of 30mT at frequency (a)-(b) 765 kHz and (c)-(d) 210 kHz. Corresponding curves for varying MNPs concentration and 1mg/mL agarose content at frequency 765 kHz and field 30mT for (e) random and (f) chain samples.*

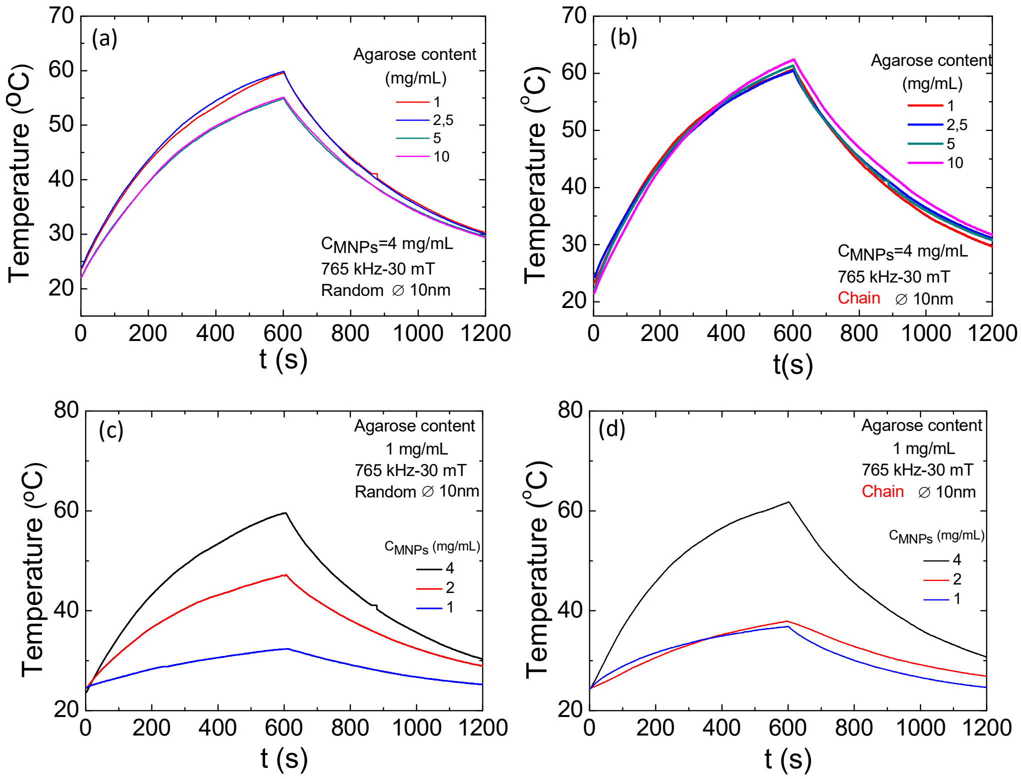

Figure S7*: Magnetic Particle Hyperthermia: Time-dependent temperature curves of 10 nm random and chain samples. Curves at field frequency of 765 kHz and amplitude of 30 mT for varying agarose content and 4 mg/mL MNPs concentration (a) chain and (b) random samples. Corresponding curves for varying MNPs concentration and 1mg/mL agarose content (c) random and (d) chain samples.*

Molecular Dynamics Algorithm

Once the basic ingredients for the model (rules for motion and interaction as described in Methods section) are defined, it is necessary to specify the algorithm for the numerical solution of the 3D model. The simulation starts from a pre-equilibrated system containing N_p MNPs (CG objects with s=1) of diameter d_0=40nm, in a volume V, and particle volume fraction $\varphi_0 = \frac{N_p}{V}\frac{\pi}{6}d_0^3$. The external magnetic field was set to 40 mT. The units of the simulation are defined by typical length and time scales. In our case, the basic unit of length is the diameter d of the colloidal particle and the basic unit of time (denoted as τ) is defined by normalizing the isotropic diffusion coefficient D of a singlet i.e. an individual non-interacting nanoparticle. Thus we convert between real units and simulation units (denoted with an upper bar) for the length and time scales by: $\bar{r} = \frac{r}{d_0}$ and $\bar{t} = \frac{t}{\tau}$ where $\tau = \frac{d_0^2}{D_1}$. As the simulation goes on, MNPs aggregate and chains with increasing values of s appear providing as outputs the number of chains containing s colloidal particles at time t, $n_s(t)$. During the simulation, we also monitor the time evolution of the average number of colloidal particles in a chain $N(t)$. To have a direct comparison with the SEM images, from the 3D computational grid, we selected similar in dimensions 2D projections, comprised of $80d_0 \times 80d_0$ (with d_0 being 40 nm in our case), solved in 10^8 steps (2400 s). The number of MNPs in the lattice was set to 380, 760, 1520 equivalent to the experimental concentrations of 1, 2 and 4 mg/mL MNPs.

Stoner-Wohlfarth model based theory for hysteresis loop estimation

With a modified Stoner-Wohlfarth model, we incorporate the role of finite temperature and frequency on the coercive field and can quantify the heating properties of MNPs systems which are far from the linear response regime. Thus we theoretically estimate the specific loss power values and directly compare them with experimental ones. Eventually, it is straightforward, that for ferromagnetic MNPs with distinct collective magnetic features, power dissipation is mainly attributed to the hysteresis losses. Ultimately, it is apparent that magnetic tuning via dipolar interactions has a direct impact on magnetic heating efficiency.

In a theoretical work dealing with the influence of a finite temperature and frequency to coercive field estimation, Usov *et al.* [2] introduced a dimensionless parameter κ for the coercive field variation taking into account the sweeping rate of the magnetic field. Mehdaoui *et al.* [3] derived an expression for the coercive field from the phenomenological fit of their numerical simulations which reads

$$\mu_0 H_c = \mu_0 H_\kappa \times \left(b - \kappa^n\right), (1S)$$

where $\mu_0 H_K$ is the anisotropy field and b and n are phenomenological constants, κ is Usov's parameter given by

$$\kappa = \frac{k_B T}{K_{eff} V} \ln\left(\frac{k_B T}{4\mu_o H_{max} M_s \tau_o V f}\right), (2S)$$

where k_B is the Boltzmann constant, T is the temperature, $\mu_0 H_{max}$ is the maximum applied magnetic field equal to 30 mT, M_s is the saturation magnetization derived from the experimental data and equal to 57 Am^2/kg, τ_0 is the frequency factor of the Néel-Brown relaxation time, V is the MNPs volume, f is the frequency and K_{eff} is the magnetocrystalline anisotropy which is approximated to 9 kJ/m^3 from relevant literature works on similar systems.[4], [5]. For our ferromagnetic MNPs, we used b=1, n=0.5, κ<0.7 as estimated by Carrey *et al.*.[6] The anisotropy field is given by:

$$\mu_o H_\kappa = \frac{2K_{eff}}{M_s}, (3S)$$

We numerically solved the problem taking into account the thermal activation of magnetization and the sweeping rate of the alternating magnetic field. By substituting equation 3 to equation 2 and then equation 2 to the differential equation 1, of the manuscript, we simulated the heating curves *T(t)* for the case of MNPs with random orientation and for the case of chain formation. With this model we can quantify the heating properties of MNPs systems which are far from the linear response regime incorporating the role of finite temperature and frequency on the coercive field. The ultimate outcome of this approach is the prediction of the specific loss power values directly comparable with experimental ones as shown in Fig. S8 with an excellent agreement between them. Thus, it is straightforward, that for ferromagnetic MNPs with distinct collective magnetic features, power dissipation is mainly attributed to the hysteresis losses. This influence be optimized with magnetic tuning via dipolar interactions having a direct impact on magnetic heating efficiency.

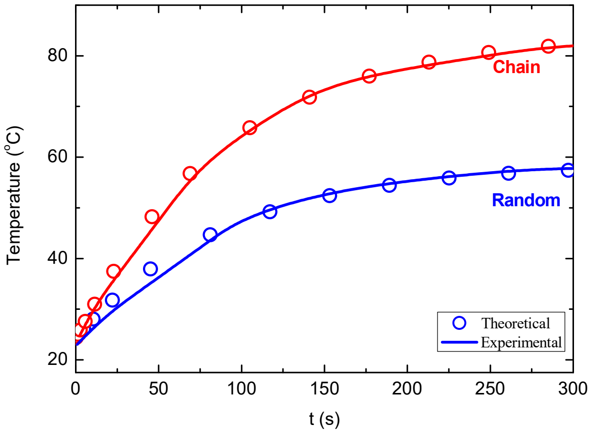

Figure S8: *Comparison between theoretical (solid lines) and experimental (symbols) heating curves for chain and random samples (40 nm MNPs, concentration 4 mg/mL and agarose content 1 mg/mL). The external field had a frequency of 765 kHz and 30 mT amplitude and was applied parallel to chain direction.*

Agarose effect

In Fig. S9a we can see that, starting from 1 mg/mL agarose content distinct SLP values are found when comparing random with chain samples. By increasing agar content,

thus solution viscosity, SLP values decrease. Solutions of agarose content ≥ 5 mg/mL, possess viscosity values that practically attenuate dipolar interactions and “block” MNPs, thus chain formation is prohibited, resulting to stable yet smaller SLP values. Similar behavior was also observed at the lower frequency of 210 kHz (Fig. S9b). As expected, this effect is practically not observed, for the 10 nm SPM MNPs, even at the high frequency (765 kHz) field (Fig. S10).

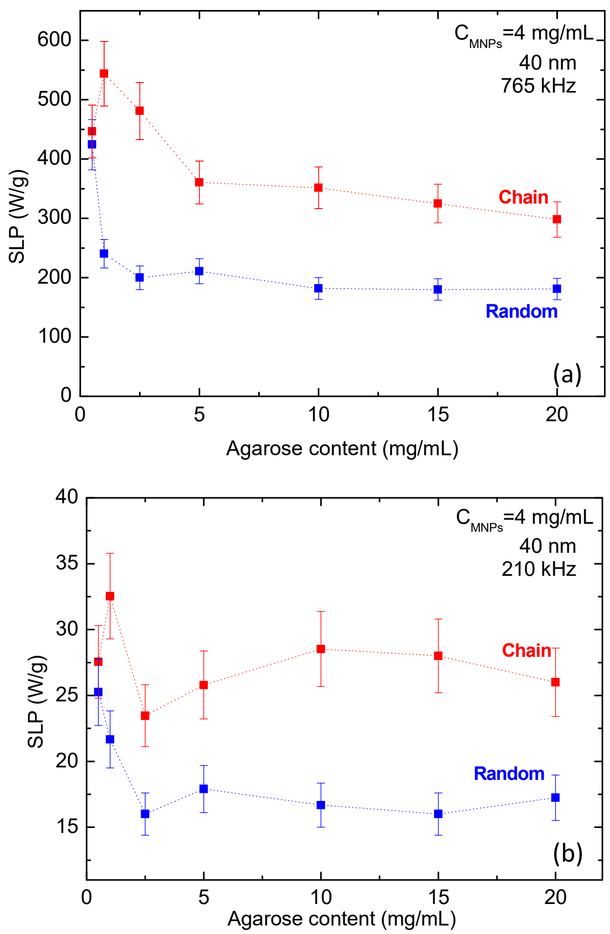

Figure S9: *Influence of agarose content on magnetic hyperthermia efficiency as expressed by SLP values for 4 mg/mL 40 nm MNPs at 30mT with frequency (a) 765 kHz and (b) 210 kHz.*

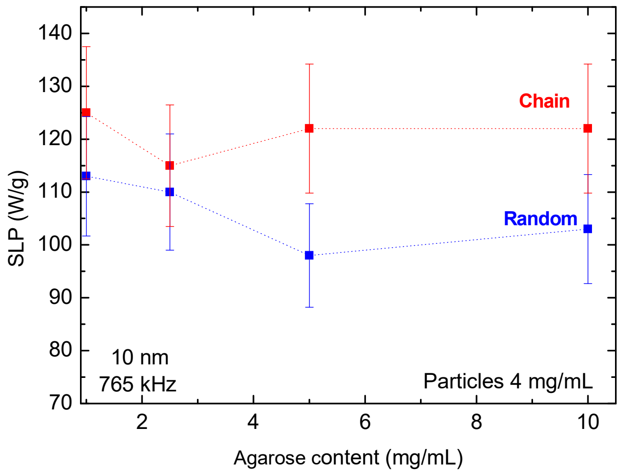

Figure S10*: Influence of agarose content on magnetic hyperthermia efficiency as expressed by SLP values for 4 mg/mL 10nm MNPs at 765 kHz and 30 mT. .*

1 Simeonidis K. et al., A versatile large-scale and green process for synthesizing magnetic nanoparticles with tunable magnetic hyperthermia features, *RSC Adv.,* **6**, 53107 (2016).

2 Usov, N.A. & Liubimov, B.Y. Dynamics of magnetic nanoparticle in a viscous liquid: Application to magnetic nanoparticle hyperthermia. *Journal of Applied Physics* **112**, (2012).

3 Mehdaoui, B. et al. Increase of magnetic hyperthermia efficiency due to dipolar interactions in low-anisotropy magnetic nanoparticles: Theoretical and experimental results. Physical Review B - Condensed Matter and Materials Physics **87**, (2013).

4 Series, S. *Magnetism in the solid state An Introduction,.* 229, (Springer Series 2006).

5 Garaio, E., Sandre, O., Collantes, J.-M., Garcia, J. A., Mornet, S. and Plazaola, F. Specific absorption rate dependence on temperature in magnetic field hyperthermia measured by dynamic hysteresis losses (ac magnetometry). *Nanotechnology*, **26(1)**, (2015).

6 Carrey, J., Mehdaoui, B. & Respaud, M. Simple models for dynamic hysteresis loop calculations of magnetic single-domain nanoparticles: Application to magnetic hyperthermia optimization. *Journal of Applied Physics* **109**, (2011).
